# Microbial biogas production from hydrolysis lignin: insight into lignin structural changes

**DOI:** 10.1186/s13068-018-1054-7

**Published:** 2018-03-09

**Authors:** Daniel Girma Mulat, Janka Dibdiakova, Svein Jarle Horn

**Affiliations:** 1 Faculty of Chemistry, Biotechnology and Food Science, Norwegian University of Life Sciences, P.O. Box 5003, 1432 Ås, Norway; 2 Norwegian Institute of Bioeconomy Research, P.O. Box 115, 1432 Ås, Norway

**Keywords:** Anaerobic digestion, Biogas, Hydrolysis lignin, Pseudo-lignin, Condensed lignin, Birch, Steam explosion, Biorefinery, Lignocellulosic biomass, NMR

## Abstract

**Background:**

The emerging cellulosic bioethanol industry will generate huge amounts of lignin-rich residues that may be converted into biogas by anaerobic digestion (AD) to increase the output of energy carriers from the biorefinery plants. The carbohydrates fraction of lignocellulosic biomass is degradable, whereas the lignin fraction is generally considered difficult to degrade during AD. The objective of this study was to investigate the feasibility of biogas production by AD from hydrolysis lignin (HL), prepared by steam explosion (SE) and enzymatic saccharification of birch. A novel nylon bag technique together with two-dimensional nuclear magnetic resonance spectroscopy, pyrolysis–gas chromatography–mass spectrometry (Py-GC/MS), and Fourier transform infrared (FTIR) spectroscopy was used to identify recalcitrant and degradable structures in the lignin during AD.

**Results:**

The HL had a lignin content of 80% which included pseudo-lignin and condensed-lignin structures resulting from the SE pretreatment. The obtained methane yield from HL was almost twofold higher than the theoretical methane from the carbohydrate fraction alone, indicating that part of the lignin was converted to methane. Characterization of the undegradable material after AD revealed a substantial loss of signals characteristic for carbohydrates and lignin–carbohydrate complexes (LCC), indicating conversion of these chemical components to methane during AD. The β-O-4′ linkage and resinol were not modified as such in AD, but major change was seen for the S/G ratio from 5.8 to 2.6, phenylcoumaran from 4.9 to 1.0%, and pseudo-lignin and condensed-lignin were clearly degraded. Scanning electron microscopy and simultaneous thermal analysis measurements demonstrated changes in morphology and thermal properties following SE pretreatment and AD. Our results showed that carbohydrate, LCC, pseudo-lignin, and condensed-lignin degradation had contributed to methane production. The energy yield for the combined ethanol production and biogas production was 8.1 MJ fuel per kg DM of substrate (4.9 MJ/kg from ethanol and 3.2 MJ/kg from methane).

**Conclusion:**

This study shows the benefit of using a novel bag technique together with advanced analytical techniques to investigate the degradation mechanisms of lignin during AD, and also points to a possible application of HL produced in cellulosic bioethanol plants.

**Electronic supplementary material:**

The online version of this article (10.1186/s13068-018-1054-7) contains supplementary material, which is available to authorized users.

## Background

In conventional first generation biorefineries based on starch and sucrose, the remaining fiber fraction is often burned to generate process heat. A similar trend is seen for the emerging second-generation cellulosic bioethanol plants, where the hydrolysis lignin (HL) is also burned to generate process heat. If the cellulosic bioethanol industry succeeds, the amount of HL produced will be very large as lignin is one of the main components of lignocellulosic biomass. For instance, the intended development of 79 billion liters of second-generation biofuels annually by 2022 in the USA would generate about 62 million tons of lignin [1], by far exceeding the current world market for lignin used in specialty products [2]. Since all these lignins are not needed for process heat, considerable efforts are underway for generating chemicals and other forms of energy from HL residues [3]. The production of high-quality oil fraction from birch HL has been reported using intermediate pyrolysis and low-cost ZSM-5 catalyst downstream process [4]. Another possible pathway is anaerobic digestion (AD) of HL into biogas, which could increase the output of fuels and improve the economic profitability of such biorefineries.

Lignocellulosic biomass consists of three major structural biopolymers, namely, cellulose, hemicelluloses, and lignin. The cellulose microfibrils are locked in a matrix of intertwined hemicelluloses and lignin called lignin–carbohydrate complex (LCC). Among the three lignocellulosic components, lignin is considered as the most recalcitrant to biological deconstruction due to its irregular, complex, and highly heterogeneous aromatic structure [3, 5].

Pretreatment is usually employed prior to biological processing for reducing biomass recalcitrance, mainly by increasing the accessibility of cellulose to enzymatic and microbial degradation. Various pretreatment methods have been applied for enhancing the digestibility of lignocellulosic biomass, including physical, chemical, biological, or combinations of these techniques [6–8]. Depending on the type of pretreatment, several characteristics of biomass are altered including biomass composition, lignin/carbohydrate structure, crystallinity, cellulose degree of polymerization, and accessibility (surface area, pore size, and pore volume) [5]. Steam explosion (SE) is considered as one of the most efficient pretreatment technologies and is one of few pretreatment techniques employed at industrial scale [9]. During SE pretreatment in a boiler, the biomass is exposed to high-pressure steam followed by a rapid decompression step, which disrupts the internal structure of lignocellulose. After SE pretreatment, enzymatic accessibility to the biomass is enhanced mainly due to opening of lignocellulosic fiber structure, solubilization of hemicelluloses, and redistribution of lignin [7].

Anaerobic digestion (AD) is a well-established technology for waste management and production of renewable energy in which organic material is converted to biogas. The conversion of complex organic compounds to biogas is possible due to the cooperation of several groups of microorganisms involved in hydrolysis, acidogenesis, acetogenesis, and methanogenesis steps [10]. Biogas consists mainly of methane (50–75%) and carbon dioxide, their relative amounts depending on the type of feedstocks and operating conditions [11]. Upgraded biogas having higher methane content can be used as a substitute of natural gas for combined heat and electricity generation, as a vehicle fuel, and can be stored in a natural gas grid for later use [11, 12]. The carbohydrate fraction of lignocellulosic biomass is degradable in AD, whereas the lignin fraction is generally considered difficult to degrade. Typically, the digestate collected from biogas digesters is rich in lignin due to the degradation of the other components [13]. However, AD of lignin has been observed in various natural environments [14–21], but studies on the fate of lignin degradation in engineered biogas systems are very limited. With a better understanding of the degradable and recalcitrant fractions of lignin during AD, potential applications beyond combustion could be developed.

In this study, biogas production from HL was investigated in AD. The chemical structure, morphology, and thermal properties of HL and the remaining undigested material after AD were characterized using a combination of advanced analytical techniques, including two-dimensional nuclear magnetic resonance (2D-NMR) spectroscopy, pyrolysis–gas chromatography–mass spectrometry (Py–GC/MS), Fourier transform infrared (FTIR) spectroscopy, scanning electron microscopy (SEM), and simultaneous thermal analysis (STA). To our knowledge, this is the first study to employ comprehensive advanced analytical techniques for showing the degradation of lignin and the changes in lignin structures during AD.

## Methods

### Inoculum and anaerobic medium

The microbial inoculum used in this experiment was collected from a full-scale continuously stirred tank reactor (CSTR) (Biowaz, Tomb, Norway) running with food waste and cow manure at mesophilic temperature (~ 37 °C). Dry matter (DM) content of the inoculum was 4.5%, the volatile solid (VS) content was 2.9%, and the pH was 7.8. This inoculum was pre-incubated anaerobically at 37 °C for 10 days to reduce endogenous biogas production. Anaerobic medium was prepared from a mixture of buffer solution, trace elements, and vitamins according to Angelidaki et al. [22].

### Raw material

Birch (*Betula pubescens*) wood chips originated from a tree harvested in 2009 in Norway (60.7°North, 10.4°East). The birch tree trunk was debarked and chipped to produce 20–30 mm chip fractions. These fractions were dried at room temperature and subsequently milled to pass a sieve of 6 mm (SM 2000, Retsch, Haan, Germany) and stored at room temperature and dry conditions. The DM and VS content of the dried birch was 94.9 and 94.8%, respectively.

### Steam explosion (SE) pretreatment

SE pretreatment was conducted using a steam explosion unit designed by Cambi AS (Asker, Norway) situated at Norwegian University of Life Science. In a previous study [23], the optimal steam explosion conditions of birch for biogas production were found to be pretreatment at 210 °C and 10 min residence time. Therefore, we used the same pretreatment conditions in this study. The non-washed steam-exploded material was stored in plastic bags at 4 °C until the start of the biogas experiment. The DM and VS content of the steam-exploded birch was 35.0 and 34.9%, respectively.

### Preparation of hydrolysis lignin (HL)

The pretreated birch was subjected to enzymatic hydrolysis using Cellic^®^ CTec2 (Novozymes, Bagsvaerd, Denmark; the protein content of the enzymatic preparation was 63.9 mg/ml). The material was added to screw-capped centrifuge tubes at a DM concentration of 10% and preheated at 50 °C before adding the enzyme. An enzyme loading of 5 mg/g DM of substrate was used, pH was adjusted to pH 5.0 by 50 mM acetate buffer, and the incubation was carried out at 130 rpm and 50 °C for 72 h to obtain a residue rich in lignin. The enzymatic reaction was stopped by heat deactivation at 100 °C for 10 min. Following deactivation, the sample was centrifuged at 10,000×*g* for 4 min, whereby the supernatant was filtered through 0.2 µm and stored at − 20 °C for later sugar analysis, whereas the solid HL was washed twice with deionized water and drying at 50 °C. The DM and VS content of the wet HL birch was 34.7 and 34.3%, respectively. The DM and VS content of the dried HL birch was 93.7 and 91.6%, respectively.

### Batch experiment to test biogas potential

The biogas production of untreated, steam-exploded, and HL birch was studied in 500 ml batch bottles. In addition, control batch bottles were prepared using inoculum alone to correct for the endogenous biogas production. All the bottles received equal amount of inoculum and anaerobic medium. The inoculum-to-substrate ratio was 2:1 (based on VS basis) as suggested by Holliger et al. [24]. The substrates were added individually into the batch bottles, while the control bottles received water instead of substrate. The bottles were flushed with nitrogen gas for 5 min and sealed with septum and aluminum caps to maintain anaerobic conditions. All the experiments were conducted in triplicate inside a shaker (Multitron Standard, Infors HT, Switzerland) at mesophilic condition (37 °C, 90 rpm). The biogas experiment run for 39 days and terminated when the daily biogas rate on 3 consecutive days was below 1% [24].

### Lignin degradation in AD using nylon bags

The degradation of HL in AD was investigated by enclosing the substrates in nylon bags. The nylon bag had a pore size of 25 µm (9 cm wide × 12 cm long, made from permeable nylon tissue, F57, ANKOM Technology, USA) which allowed the contact between the anaerobic microbes and the substrate enclosed by the bag. This technique is commonly employed for evaluating the in vivo digestibility of animal feed in the rumen of fistulated animals [25, 26].

The nylon bag was first weighed and the desired amount of substrate (about 1.0 g DM) was transferred into the bag and heat-sealed afterwards. The sealed bags were added into the serum bottles containing inoculum and anaerobic medium. The headspace of the bottles was flushed with nitrogen gas for 5 min to maintain anaerobic conditions and then sealed with septum and aluminum caps. All the experiments were conducted in triplicate in shaker at 37 °C as described above. After 39 days of AD, all nylon bags were collected and washed under running water and dried in an oven at 50 °C. The dried bag was opened to collect the undigested substrate for further characterization of the material with advanced analytical techniques such as NMR, FTIR, Py–GC/MS, STA, and SEM as described below. The nylon bags from triplicate bottles were collected and the undegradable materials remaining inside the nylon bags were pooled together.

### ^1^H-^13^C heteronuclear single quantum coherence (HSQC) nuclear magnetic resonance (NMR) analysis

The 2D HSQC NMR spectra were recorded on a Bruker AVIII 400 MHz spectrometer at 25 °C. About 50 mg of sample was dissolved in 0.75 ml of DMSO-*d*_6_ (99.8% D). Bruker’s “hsqcetgpsi2” pulse program with 5000 Hz (from 10 to 0 ppm) and 20,843 Hz (165 to 0 ppm) for the ^1^H- and ^13^C-dimensions, respectively, was used. The number of collected complex points was 2 K for the ^1^H dimension with a recycle delay of 5 s. The number of transients was 120, and 256 time increments were always recorded in the ^13^C dimension. ^1^J_C–H_ used was 145 Hz. Processing used typical matched Gaussian apodization in the ^1^H dimension and squared cosine-bell apodization in the ^13^C dimension. Prior to Fourier transformation, the data matrixes were zero filled up to 1024 points in the ^13^C dimension. The central solvent peak was used as an internal reference (*δ*_C_ 39.5; *δ*_H_ 2.49). HSQC correlation peaks were assigned by comparison with the literatures [27–29]. A semi-quantitative analysis [27] of the volume integrals (uncorrected) of the HSQC correlation peaks was performed using standard Bruker Topspin 2.1 NMR software. In the aliphatic oxygenated region, the relative abundances of side chains involved in the various inter-unit linkages were estimated from the C_α_–H_α_ correlations to avoid possible interference from homonuclear ^1^H–^1^H couplings. In the aromatic/unsaturated region, H_2,6_ and S_2,6_ correlations from *p*-hydroxyphenyl (H) and syringyl (S) lignin units, respectively, and G_2_ correlation from guaiacyl (G) were used to estimate their relative abundances [27]. For semi-quantitative calculation, part of the aromatic region is defined as internal standard and the amount of linkages and units are expressed as a number per 100 aromatic units (H+G+S). Half of the volume integral of H_2,6_ and S_2,6_ correlation peak was used as the area of the H_2,6_ and S_2,6_ correlation peaks corresponds to twice the amount of H and S units, respectively (i.e., H_2,6_/S_2,6_ peak contains H_2_/S_2_ and H_6_/S_6_ correlations). The integral value obtained for the H_2,6_/2+S_2,6_/2+G_2_ is then set to 100Ar.

### Fourier transform infrared (FTIR) spectroscopy analysis

The FTIR spectra were obtained on a Nicolet iS50 FTIR spectrophotometer (Thermo Scientific, USA). Each sample was deposited uniformly on the surface of the glass disc and the FTIR spectra was acquired against pre-established background by averaging 64 scans from 4000 to 650 cm^−1^ at 4 cm^−1^ resolution.

### Pyrolysis–gas chromatography–mass spectrometry (Py–GC/MS) analysis

Py–GC/MS measurements were performed with a filament pulse pyrolyzer (Pyrola2000, PyrolAB, Sweden), which was connected to a GC/MS instrument (7000C Triple Quadrupole GC/MS System; Agilent Technologies, Inc., Bellevue, WA, USA). About 100 μg of the sample was placed directly on the Pt filament, which contained a small cavity. Pyrolysis chamber maintained at 175 °C was purged with Helium 18 ml/min to lead the pyrolysis products into the gas chromatography injector, which contained split liner (Restek, 3.4 mm × 5.0 mm × 54 mm). The instrument (Pyrola 2000) is capable of fast pyrolysis with a heating rate of 175 °C/ms. Temperature rise time to the final pyrolysis temperature 600 °C was set to 8 ms and total pyrolysis time was 2 s. Pyrolysis products were separated using a capillary column (TraceGOLD TG-1701MS, 60 m × 0.25 mm i.d, 0.25 μm film thickness; ThermoFisher Scientific), using the following temperature program: 4 min at 50 °C, 5 °C/min to 130 °C, 4 min hold at 130 °C, 2 °C/min to 170 °C, 4 min hold at 170 °C, 5 °C/min to 270 °C, and 4 min hold at 270 °C. Helium was used as a carrier gas, using constant flow rate of 1.0 ml/min. The pyrolysis interface, injector, detector, and transfer line temperatures were kept at 201, 250, 250, and 250 °C, respectively. The MS was operated in EI mode with 70 eV electron and with full scan mode between *m*/*z* 40 and 720. Average of at least two measurements was calculated and the peak areas of the pyrolysis products were normalized to 100%. The pyrolysis products formed were identified using standard samples, data from the literature [30–33], and commercial NIST 11 MS library.

### Simultaneous thermal analysis (STA)

STA was used to investigate the thermal behavior and chemical composition of all investigated samples. The experiments were set up on a Simultaneous Thermal Analyzer apparatus (STA, Netzsch 449 F1 Jupiter) with the enthalpy determination accuracy of 3%. About 6 mg of each birch powder and reference material were placed into separate Al_2_O_3_ crucibles (0.3 ml volume) without lid and were put into the silicon carbide furnace sample holder while heating at a constant heating rate of 10 K/min from 40 to 600 °C. The experiment was performed under an oxidizing atmosphere (N_2_/O_2_ 80:20 vol.%) [34, 35]. The experiment was conducted at atmospheric pressure and using a volume flow rate of 20 ml/min. A synthetic air, used as purged and protective gas, was allowed to flow into the apparatus prior to each run, where the volume flow rate was maintained constant at 20 ml/min and atmospheric pressure. The temperature difference of samples was measured at heating rate of 10 K/min. After each single sample measurement, the furnace was cooled down to ambient temperature to get ready for the next run. The experimental measurements of all samples were repeated three times. Thermogravimetric (TG) and Gram Schmidt (GS) curves of samples were corrected by the baseline obtained from runs with empty crucibles.

### Acid-insoluble lignin and carbohydrate analysis

Samples for carbohydrate and acid-insoluble lignin content analysis were prepared using a standard NREL two-stage acid hydrolysis protocol [36]. Acid hydrolysis generates soluble sugars and acid-insoluble lignin residues, where the later was oven-dried and weighed to obtain the acid-insoluble lignin (Klason lignin) content. The soluble sugars were analyzed for carbohydrate constituents by high-performance anion-exchange chromatography with pulsed amperometric detection (HPAEC-PAD) using Dionex ICS-3000 (Dionex Corp., USA).

### Biogas composition and calculation

The biogas production was periodically monitored by measuring the gas pressure in the headspace of the batch bottles using a digital pressure transducer (GMH 3161, Greisinger Electronic, Germany). After recording the pressure in the batch bottles, the overpressure was released by penetrating the septum with a needle. To avoid excessive dissolution of CO_2_ with possible effects on pH, the overpressure was always kept below 200 kPa [24]. The biogas composition (CH_4_ and CO_2_) was analyzed by gas chromatography (GC) using a gas chromatograph (3000 Micro GC, Agilent Technologies, USA) equipped with a thermal conductivity detector (TCD). Using the measured overpressure, headspace volume of the bottles and measurements of methane concentrations as input, the ideal gas law was applied for calculating methane production. The methane production was reported at standard temperature and pressure (0 °C and 1 atm.) after correcting the background methane production from inoculum alone (control). The average results of the biological triplicates are presented with standard deviations.

### Other analytical methods

Dry matter (DM) and volatile solids (VS) of substrates were analyzed according to the standard methods [37].

### Scanning electron microscopy (SEM) analysis

After mounting dry samples on aluminum specimen stubs and sputter coating with gold, SEM images of birch samples were acquired on a Zeiss EVO 50VP (Cambridge, UK) SEM at 15 kV beam accelerating voltage and various resolving powers. Several pictures were taken at different magnifications (200× to 15,000× magnification) for each sample surface. First, low magnification was applied to obtain an overview of the sample surface’s topographical features and morphology, and then, pictures were taken at higher magnification, focusing on typical surface features.

## Results and discussion

### Substrates characteristics

The DM, VS, and pH of the untreated, pretreated and HL birch samples differed considerably. The DM content decreased from 94.8% in untreated birch to 35.0% in steam-exploded birch as steam is added to the biomass during pretreatment. The washed and centrifuged HL had a DM content of 34.7%. In comparison with the VS content of 99.8% (DM basis) for untreated and pretreated birch, the VS of HL birch was slightly lower (98.7%), indicating an enrichment of ashes in the HL. The pH of steam-exploded birch was 3.0, which was lower than the pH 5.0 of HL birch. The low pH can be explained by the release of organic acids from the degradation of hemicelluloses during SE pretreatment [38]. The elevated pH of HL is the result of pH adjustment to 5.0 before the enzymatic hydrolysis. The pH of the HL residue and steam-exploded material was corrected to 7–8 prior to the biogas experiments.

The content of cellulose, hemicelluloses, and Klason lignin in the three birch samples is summarized in Table 1. The proportion of cellulose and Klason lignin in the steam-exploded birch increased, while the amount of hemicelluloses (mainly xylan) was reduced. Such large reductions in the content of hemicelluloses have been reported in a previous study of birch pretreated with SE [23]. The employed high temperature and acidic conditions (released organic acids) during SE can catalyze the hydrolysis of hemicelluloses and further degradation into lower molecular weight (LMW) compounds such as furfural. These LMW compounds may undergo polymerization reactions, forming a lignin-like material termed “pseudo-lignin” [39]. Thus, formation of pseudo-lignin was probably the main reason for the increase in the lignin fraction after pretreatment, although loss of volatiles during SE formed from hemicellulose would also contribute to an increase in the lignin and cellulose fraction. It should be noted that the SE material was not washed and represents the whole slurry obtained after the pretreatment. Pseudo-lignin has been shown to be detrimental to cellulosic enzymatic hydrolysis of cellulose [40], but its fate in AD is unknown. During enzymatic saccharification, pseudo-lignin deposits on polysaccharides could lead to non-productive binding of enzymes and acts as a barrier to accessing cellulose and thereby reducing cellulose breakdown [40]. After enzymatic hydrolysis of the pretreated material, the carbohydrate fraction of HL birch was substantially reduced to 10% and the Klason lignin increased accordingly to 80%. The obtained sugar yield after pretreatment was 90% of the theoretical sugar expected. The remaining 10% carbohydrates and part of the lignin fraction in the HL birch could be potential substrates for methane production.

**Table 1:** Chemical composition of different birch samples

Birch	Composition (%)					
	Glucan^b^	Arabinan	Galactan	Xylan	Mannan	Klason lignin
Untreated	36.5 ± 2.04	1.1 ± 0.02	1.4 ± 0.04	16.5 ± 0.42	2.8 ± 0.12	29.2 ± 1.12
Steam-exploded^a^	43.2 ± 2.06	0.3 ± 0.01	0.9 ± 0.04	9.9 ± 0.08	1.7 ± 0.02	40.3 ± 1.02
HL	9.7 ± 1.01		0.1 ± 0.01	1 ± 0.02	0.6 ± 0.01	79.8 ± 3.27

The amounts of all components are expressed as a percentage of dry matter. The amount of carbohydrates was calculated using the mass of anhydrous sugar

^a^The SE material was not washed and represents the whole slurry obtained after the pretreatment

^b^It should be noted that while almost all of the glucan originate from cellulose, hemicelluloses also contain some glucose that will contribute to the glucan fraction

### Biogas potential of hydrolysis lignin

**Fig. 1:**
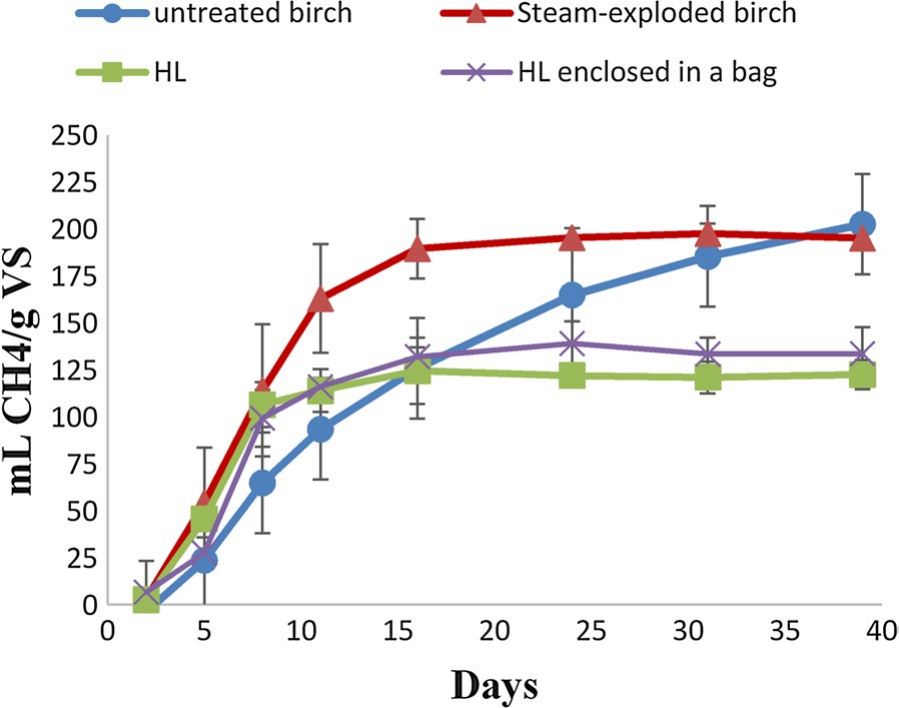
Cumulative methane production of birch samples. Untreated (circles), steam-exploded (triangles), HL (squares), and HL birch enclosed in a nylon bag (crosses)

Figure 1 shows the cumulative methane yield of untreated, pretreated, and HL birch monitored for 39 days. The initial rate of methane production of pretreated and HL birch was higher than untreated birch, indicating an increased accessibility of the birch material to anaerobic bacteria after SE pretreatment. While the steam-exploded birch reached maximum methane yield after 16 days, the untreated material needed 39 days to reach similar yields. In this study, the main effect of SE pretreatment is on the rate of methane production, not on the final yield. Previous studies have shown that SE can increase the initial methane production rate, but that the increase in methane yield depends primarily on the type of substrate and severity factor (function of temperature and residence time). The methane yield increased from 291 ml/g VS in untreated rape straw to 257 ml/g VS in SE pretreated straw at higher severity factor (230 °C, 10 min) [41]. The initial methane production rate also increased with an increase in the severity of pretreatment (i.e., with increasing time and temperature). In another study of a sugarcane bagasse subjected to SE pretreatment, the methane production rate was improved substantially and methane yield was increased by 1.3 times than the yield from untreated bagasse [42]. In another batch biogas experiment with wheat straw steam-exploded at three different conditions (180, 200, and 220 °C for the same residence time of 15 min), the methane yield significantly increased for only the pretreated material at 180 °C [43].

Figure 1 shows that the methane potential of HL residues originated after enzymatic removal of the carbohydrates. The final methane yield of HL birch was 125 ml CH_4_/g VS, reached after 16 days of incubation. Considering the carbohydrate composition of HL birch (Table 1) and a theoretical methane yield of 415 ml/g DM of carbohydrates, about 47 ml of methane could be expected to be produced from the carbohydrate fraction. The observed methane yield of 125 ml CH_4_/g VS from HL birch must mean that part of the lignin fraction of HL also was converted into methane. There are few studies on biogas production of lignin-rich residues in a biorefinery setting. Batch biogas bottles fed with lignin-rich residues of hemp showed that the methane yield was similar to untreated hemp (i.e., 127 ml CH_4_/g VS) [44]. This lignin-rich material was a residue remaining after distillation of bioethanol from fermentation broth. The hemp was pretreated by SE (210 °C for 5 min after impregnation with 2% SO_2_) to improve the bioethanol production during simultaneous saccharification and fermentation. It should be noted that residual bioethanol, the enzyme solutions, yeast, and yeast extract all contain carbon sources and can contribute to methane production [44]. In another study, alkali-pretreated sugarcane bagasse was used for combined bioethanol and biogas production based on a high-solid fed-batch SSF (simultaneous saccharification and fermentation) process with delayed inoculation (DSSF) [45]. The lignin-rich residue after evaporation of the bioethanol gave a high methane yield of 307 ml CH_4_/g VS compared to our study and others [44]. The high methane yield could be due to a high content of processing residues such as yeast and residual bioethanol.

**Fig. 2:**
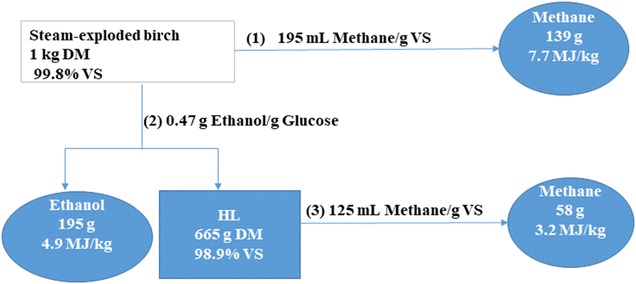
Process scheme for methane and bioethanol production from birch. Methane production from steam-exploded birch (1), ethanol production from steam-exploded birch (2), and methane production from HL residue (3). The combined ethanol and methane production is represented by combination of (2) and (3)

The energy content of produced methane and theoretically produced ethanol was calculated (Fig. 2) to evaluate the energy recovery from only methane production (process no. 1), only ethanol production (process no. 2), and combined ethanol and methane production (process no. 2 and 3). In a combined ethanol and methane production, ethanol is produced from the steam-exploded material and the methane from the HL residue. For calculations, 26.8 and 55.7 MJ/kg were used as the high heating values (HHV) of ethanol and methane, respectively. Our group previously used the same substrate and obtained a maximum ethanol yield of 0.47 kg/kg of glucose for substrate loading of 10% w/w was used [46]. The combined ethanol and methane production gave an energy yield of 8.1 MJ fuel per kg DM of substrate (4.9 MJ/kg from ethanol and 3.2 MJ/kg from methane), which is higher than the yield from only methane production (7.7 MJ/kg) or only ethanol production (4.9 MJ/kg). For steam-exploded sugarcane bagasse, the combined process gave 7.1 MJ/kg of energy, whereas only 4.1 MJ/kg of energy was obtained from bioethanol production alone [42]. In another experiment with steam-exploded oat straw, 9.5 MJ/kg of energy was achieved for the combined process and 7.4 MJ/kg for methane alone [47]. Thus, utilization of lignin-rich residues after bioethanol production for production of biogas may clearly increase the output of energy carriers from lignocellulosic biomass.

### Nylon bag technique optimization

The nylon bag technique was originally developed for evaluating the digestibility of animal feed [26], but its use in engineered biogas digesters is very limited. This technique is widely used for in vivo degradation studies, where animal feed enclosed in a bag is directly introduced into a rumen of a living animal through fistulas before finally removed and washed for subsequent chemical analysis [25, 26]. Given the similarity of rumen system and biogas digesters, we adopted this technique for evaluating degradation of HL in biogas digesters.

The main purpose of using of the nylon bag technique was to collect a clean material of the undigested substrate after AD, which would be suitable for further chemical and structural analysis. It should be noted that the inoculum, which originated from a food waste and manure treating biogas plant, contained some fiber material that would contaminate the lignin-rich birch if it was not enclosed in a nylon bag. With the use of an appropriate pore size of the nylon bags, we retained the lignin substrate inside the bag and allowed contact between the substrate and microbes while avoiding contamination of the substrate with the fibers originating from the inoculum (see the discussion below). Since microbes can get in and out of the bags, the bags were washed with running water to remove the microbes prior to harvesting the undigested lignin.

AD of HL enclosed in a nylon bag gave very similar methane production rate and yield as the one without nylon bag (Fig. 1), showing that the nylon bag does not influence the AD process. Use of nylon bags may affect methane production in CSTRs operated for long periods, since the nylon bags could create a surface conducive for the establishment of microbial biofilms [48, 49]. This may affect the accessibility of the substrates to microorganisms, provide closer interspecies distance between bacterial and methanogens, and increase the retention time of the microbes in the reactor. Our results showed that the use of nylon bag did not influence AD process, which could be due to the short operating period in the batch experiments reducing microbial biofilm formation.

A nylon bag with low porosity is needed to reduce substrate leak and contamination of the undigested substrate with fiber materials originating from inoculum, while at the same time permeable for the anaerobic microbes. Moreover, the amount of substrates enclosed in nylon bags should not be increased to the level that physically reduces the contact between microbes and substrate. The similarity of methane production of HL with and without nylon bag, together with the acquired high-quality chemical data of the undigested substrate (see the discussion in next section), confirmed that enclosing about 1.0 g DM of HL in the nylon bags (9 cm wide × 12 cm long, a porosity of 25 µm) was an appropriate amount.

### Insight into lignin structural changes after pretreatment and AD

#### NMR analysis

**Fig. 3:**
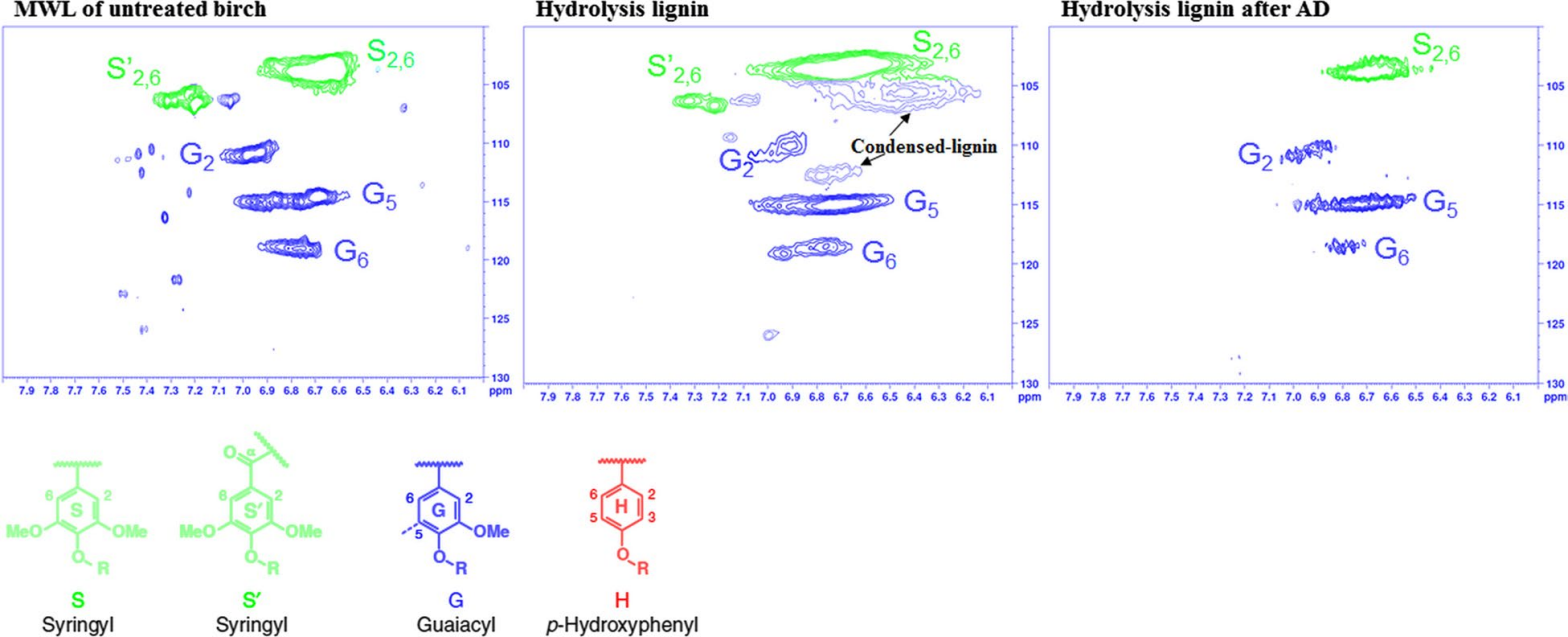
The 2D HSQC NMR spectral regions of interest (aromatic region) for birch samples and the structures of main lignin units (S, G, and H). Numbers indicate carbon atoms in the aromatic ring. For well-resolved correlation peaks, contours are color coded to match their structures shown

**Fig. 4:**
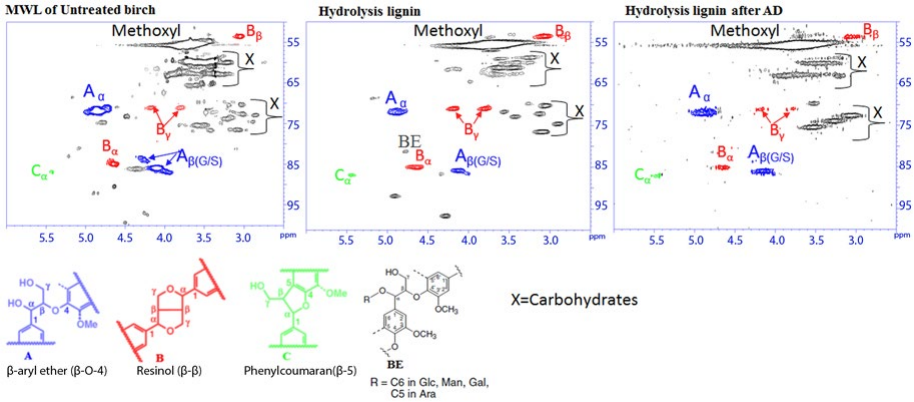
The 2D HSQC NMR spectral regions of interest (aliphatic region) for birch samples and the structures of lignin inter-unit linkages. The letters found in the spectra refer to the substructures shown below the spectra. For well-resolved correlation peaks, contours are color coded to match their structures shown

The HSQC spectral regions of interest for untreated birch MWL, birch HL before and after AD as well as the structures of the main lignin units and lignin inter-unit linkages are shown in Figs. 3 and 4, respectively. The regions displayed include the aromatic structures of lignin (Fig. 3) and the aliphatic lignin-side chains (Fig. 4). The relative abundance of different inter-unit linkages per 100Ar, S units, G units, and S/G ratios is shown in Table 2. Detail assignments of correlation peaks are given in Additional file 1: Table S1. The HSQC NMR spectrum of MWL prepared from untreated birch, representing native lignin in birch [28] showed that birch is a typical hardwood (S/G-type lignin) with substantial amounts of S units, G units, lignin methoxyls and an S/G ratio of 2.8 (Fig. 3 and Table 2). The lignin-side-chain correlations (Fig. 4) for untreated birch MWL show that β-O-4′ was the major lignin inter-unit linkage (67%, substructure A), followed by resinol units (13%, substructure B) and phenylcoumaran units (2.6%, substructure C). Our results are comparable with a previous HSQC NMR study of birch reaching 2.0 S/G, 69% β-O-4′, 17% resinol, and 3% phenylcoumaran [50].

**Table 2:** Abundance of major lignin inter-unit linkages and S/G for birch samples

Materials	Amounts per 100Ar (%)				Weight (%)		S/G
	β-O-4′	β–β	β-5	BE	S	G	
MWL of untreated birch	66.9	13.3	2.6	BD^a^	74.0	26.0	2.8
HL birch	18.6	11.0	4.9	3.9	85.0	15.0	5.8
HL birch after AD	23.5	9.6	1.0	BD	72.0	28.0	2.6

^a^BD means below detection limit

The change in lignin structure after SE pretreatment was investigated by comparing the HSQC spectra between untreated birch MWL and birch HL (Figs. 3, 4, and Table 2). The β-O-4′ linkage was substantially reduced from 67% in untreated birch to 18% in HL, showing the cleavage of this major lignin linkage by SE pretreatment. The correlation peak of S_2,6_ at δC/δH 105/6.6 ppm became broad due to overlap with other signal, which was not observed in untreated birch spectra. Therefore, the calculated S/G ratio increased from 2.8 in untreated birch to 5.8 in HL as the C–H correlation of S_2,6_ at δC/δH 105/6.6 increased due to signal overlap. An additional C–H correlation appeared at δC/δH 112.3/6.75 ppm in HL, which has not been reported previously. These new signals could be associated with the formation of pseudo-lignin in HL, as it has a lignin-like aromatic structure [40, 51]. It could also be associated with modification of aromatic ring in native lignin, resulting in a formation of condensed-lignin [38, 52, 53]. A condensed-G-type lignin results from a new bond formed between the G_5_ and/or G_6_ positions of G units and other lignin unit [53]. While the exact structures of condensed lignin formed in S units remained unknown. It is hypothesized that the condensed position in S units is probably located at C_α_ of the side chain, which forms a new C–C bond between an aromatic ring and a reactive carbonium ion, normally located at C_α_ of the side chain [54]. Moreover, the decrease in G_6_/G_5_ ratio from 0.5 to 0.3 after SE pretreatment suggests that the formation of condensed lignin is higher at the G_6_ position of G units as reported previously [53]. G_6_ has higher potential to participate in condensation reactions, since it is para to the methoxy group. It should be noted that the HSQC signals are originated from the non-substituted C–H bonded aromatic positions in condensed-lignin structures, whereas the new C–C-bonded aromatic positions do not produce cross correlations.

The changes in lignin structure after SE pretreatment also clearly shown by changes in other lignin-side changes. Notably, the signal intensity of phenylcoumaran increased after SE, which could be due to formation of new phenylcoumaran bond following the cleavage of β-O-4′ linkages as demonstrated by Heikkinen et al. [52]. The intensity of resinol signals was not significantly changed after SE pretreatment, indicating the resistance of this bond to modification by SE pretreatment. The presence of minor amount of benzyl ether (BE, 4%) in birch HL and its absence in the untreated birch MWL could indicate limitations in the MWL method for isolating lignin containing the BE substructure. Our results are in agreement with a previous study [28], where cellulosic enzymatic hydrolysis and MWL lignin isolation methods were the best and worst, respectively, to study BE lignin–carbohydrate complex (LCC) linkages. Over all, the HSQC NMR results show the modification of lignin structure after SE, particularly the simultaneous cleavage of β-O-4′ aryl-ether linkages and re-condensation to form β-5′ linkages and other condensed-lignin structures.

On day 39, the nylon bags were removed from the biogas digesters running with HL to characterize the chemical structure, morphology, and thermal properties of the remaining solid material by NMR, Py–GC/MS, FTIR, SEM, and STA methods. The HSQC spectra of the original HL substrate and the remaining HL after AD are shown in Figs. 3 and 4. In comparison with the original HL substrate, the S/G ratio of the material after AD was reduced to 2.6 (Table 2) and the S_2,6_ correlation at δC/δH 105/6.6 ppm became as narrow as the untreated birch MWL. These results in conjunction with the absence of correlation at δC/δH 112.3/6.75 ppm indicate the degradation of pseudo-lignin and/or condensed-lignin during AD. The proportion of phenylcoumaran and BE was reduced during AD from 5 to 1%, and from 4% to below detection limit, respectively, whereas the proportion of β-O-4′ and resinol did not change. Degradation of BE linkage of lignin in AD by ruminal microbes has been reported previously [55], but the degradation of phenylcoumaran linkage of lignin has not been reported. The cleavage of LCC like BE may be due to the solubilization of the carbohydrate component. As expected, the HSQC NMR results show that polysaccharide signals were diminished substantially after AD (Fig. 4).

#### FTIR analysis

FTIR spectroscopy analysis was used to further investigate changes in the chemical structure of birch samples caused by SE and AD (Additional file 1: Figure S1). All birch samples exhibited the characteristic bands of cellulose including C–H-stretching vibration in aliphatic (2922 cm^−1^), the C–O–C symmetric and antisymmetric stretching (1370 and 1162 cm^−1^, respectively) and C–O valence vibrations (1015–1060 cm^−1^) as well as vibration of the aromatic ring (1600–1616, 1515 cm^−1^), C–H-bending vibration in methyl groups (1460–1470 cm^−1^) and a broad band between 3000 and 3600 cm^−1^ representing –OH-stretching vibrations [56–58]. The band at 1716–1733 cm^−1^ in untreated (Additional file 1: Figure S1A) and pretreated (Additional file 1: Figure S1B) samples is typical C=O stretching in hemicelluloses [59] and/or pseudo-lignin [40, 51]. Despite the loss of some of the hemicelluloses after pretreatment (Table 1), a higher intensity of the band at 1716–1733 cm^−1^ was seen for the pretreated sample suggesting the formation of pseudo-lignin after SE pretreatment. The higher intensity of a signal at 1615–1600 cm^−1^ after pretreatment, representing C=O conjugated to aromatic rings [56], may indicate formation of a pseudo-lignin, which is known to having carbonyl functional group [40, 51]. It should be noted that the structures of the Hibbert ketone type originating from the cleavage of β-O-4′ are unconjugated ketones as C=O is located at C_β_ position [53]. It was not possible to confirm the depolymerization of β-O-4′ using FTIR because of the bands attributed to C_alkyl_–O ether vibrations (methoxyl and β-O-4′) at 1047–1004 cm^−1^ region overlaps with the cellulose C–O valence vibrations in the range of 1060–1015 cm^−1^. SE pretreatment also led to reduction in intensity of a band at 1162 cm^−1^ representing C–O–C asymmetric valence vibration in hemicellulose [60]. Additional bands appeared at 1327 and 1114 cm^−1^ in steam-exploded birch, showing aromatic ring C–O and C–H in-plane deformation in S lignin units, respectively [61]. These signals could also relate to condensation of G units at position G_5 [_61_]_, which has similar structure as S units.

The FTIR analysis further demonstrated the removal of carbohydrates and enrichment of the lignin fraction in the HL after enzymatic hydrolysis of the pretreated birch (Additional file 1: Figure S1C). In comparison with the steam-exploded birch, the appearance of an additional weak band in HL at 880 cm^−1^, representing the aromatic C–H out-of-plane bending, is due to the enrichment of lignin after enzymatic hydrolysis. The removal of cellulose was shown as reduction in intensities of the C–O stretching at 1240 cm^−1^ and the C–O valence vibration at 1045 cm^−1^ in HL. Because of cellulose removal, the weak bands at 1214 cm^−1^ (C–C and C–O stretch in G condensed) and 1114 cm^−1^ (C–H stretch in S unit and/or condensed-G units at position G_5_) [61] became more strong in HL. This is consistent with the observed lignin structural modification seen by the NMR analysis.

The HL birch after AD (Additional file 1: Figure S1D) showed similar profiles as the original HL material, but lower intensities were observed for the bands at 1735–1710 (C=O stretching in hemicelluloses and/or pseudo-lignin), 1325 (aromatic ring C–O in S units and/or condensed-G units at position G_5_), 1214 (C–C and C–O stretch in G condensed), and 1114 cm^−1^ (aromatic ring C–H stretch in S unit and/or condensed-G units at position G_5_) in the former. The reduction in intensity of these bands is consistent with the degradation of condensed-/pseudo-lignin and removal of hemicelluloses shown by the HSQC NMR analysis.

#### Py–GC/MS analysis

**Fig. 5:**
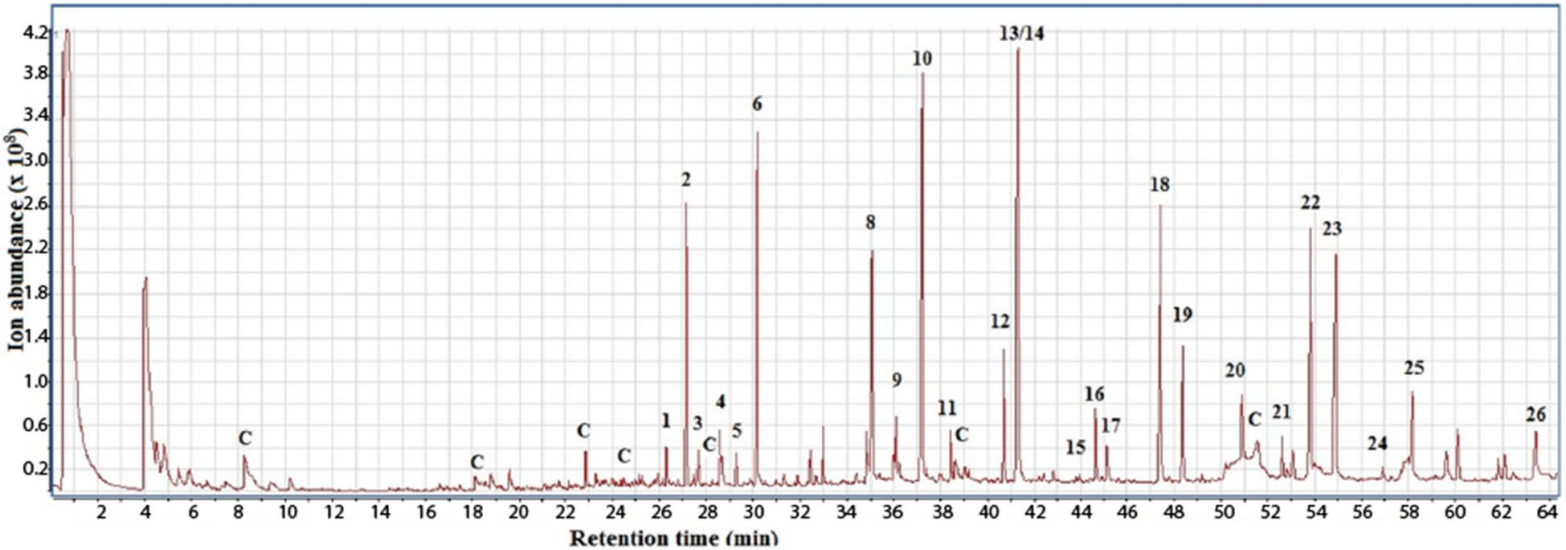
Pyrograms of HL birch obtained at 600 °C. Peak identities and relative abundances of pyrolysis compounds released from HL birch and other birch samples are listed in Table 3

Py–GC/MS analysis was applied to all the birch samples. As an example, the pyrogram of HL is shown in Fig. 5, illustrating the well-separated pyrolysis products derived from the lignin and carbohydrate components of HL birch. Similar pyrograms were produced and identified for all samples giving a total of 27 identified lignin pyrolysis products, including 4 H units, 11 G units, and 12 S units (Table 3). The relative proportion of lignin-derived pyrolysis products was calculated to determine the difference in lignin structure of the various birch samples (Table 3). The peaks marked with “C” in the pyrograms originated from cellulose and hemicelluloses degradation (e.g., 5-HMF, furfural, and levoglucosan) were not included in the calculations. In addition to the untreated and steam-exploded birch, the lignin-rich fractions (HL before and after AD) also gave rise to small amounts of carbohydrate-derived pyrolysis compounds.

**Table 3:** Relative abundance of lignin-derived pyrolysis products of birch samples at 600 °C

Pyrolysis products	Peak no.	RT (min)	Relative abundance (%)^a^			
			Untreated birch	Steam-exploded birch	HL	HL after AD
Phenol	1	26.303	1.0	1.4	0.8	2.4
Guaicol	2	27.141	3.8	6.9	6.1	8.8
Phenol,2-methyl	3	27.658	0.6	0.9	0.8	1.3
*p*-Cresol	4	28.417	3.7	1.6	0.7	1.8
Phenol,4-methoxy-3-methyl-	5	29.285	1.3	0.7	0.8	0.8
Phenol,2-methoxy-4-methyl-	6	30.149	4.2	7.0	9.0	12.8
Phenol,4-ethyl-2-methoxy-	7	32.967	2.7	1.1	1.5	2.1
2-Methoxy-4-vinylphenol	8	35.035	5.2	6.5	6.2	10.9
Eugenol *m*/*z* 164	9	36.085	0.3	0.2	0.2	0.7
Phenol,2,6-dimethoxy-	10	37.169	10.9	17.4	15.4	8.0
Phenol,2-methoxy-5-(1-propenyl),(*E*)-	11	38.418	1.3	1.1	1.6	2.5
*trans*-Isoeugenol	12	40.67	4.1	3.3	4.3	8.7
1,2,4-Trimethoxybenzene 168 *m*/*z*	13	41.218	1.8	3.1	4.0	2.2
Vanillin 152 *m*/*z*	14	41.297	1.5	0.8	0.9	1.7
Phenol,2-methoxy-4-propyl-	15	43.989	2.4	1.0	0.0	0.8
5-*tert*-Butylpyrogallol	16	44.665	1.2	1.2	2.4	0.9
Acetovanillone	17	45.148	2.4	0.9	1.4	3.4
3,5-Dimethoxyacetophenone	18	47.385	11.1	11.6	11.2	9.1
Phenol,2,6-dimethoxy-4-(2-propenyl)-^b^	19	48.361	4.4	3.8	4.8	3.3
Phenol,2,6-dimethoxy-4-(2-propenyl)-^b^	20	50.915	1.6	1.2	2.2	1.4
2-(2,5-Dimethoxy-4-ethylphenyl)ethylamine, *N*-acetyl-	21	52.649	1.4	1.2	1.4	0.9
Phenol,2,6-dimethoxy-4-(2-propenyl)-^b^	22	53.788	9.2	7.3	9.5	7.1
Syringaldehyde	23	54.833	12.0	9.9	10.3	4.8
Homosyringic acid	24	56.93	5.4	2.7	0.0	0.0
Acetosyringone	25	58.173	4.4	3.7	2.3	2.3
Sinapyl alcohol	26	60.123	1.2	3.1	1.9	1.3
Sinapic aldehyde	27	63.501	0.9	0.4	0.2	0.2
Total			100.0	100.0	100.0	100.0
H (%)			5.5	3.7	4.0	4.0
G (%)			29.3	29.4	31.9	53.1
S (%)			64.3	65.5	63.4	40.5
S/G ratio			2.2	2.2	2.0	0.8
Ph-C1,C2/Ph-C3 ratio			0.7	0.9	0.9	1.2
Oxygenated side chain (%)			40.4	34.4	29.6	23.7

^a^The peak areas of the lignin-derived pyrolysis products were normalized to 100%

^b^Peaks 19, 20 and 22 are all assigned as the same compound by the NIST 11 MS library, and are likely isomers

As shown in Table 3, the main lignin pyrolysis products originating from untreated birch hardwood were S and G units with an S/G ratio of 2.2. In comparison with NMR analysis, the S/G ratio was slightly underestimated by Py–GC/MS. Our results are consistent with a previous study, where the S/G ratio in hardwood determined by Py–GC/MS was lower, possibly due to enhanced demethoxylation of S-type units during thermolysis [62]. The proportion of H type units reached less than 6% of all lignin-derived pyrolysis products, and was not detected by the NMR analysis. This difference is expected due to the higher sensitivity of mass spectrometry and the possibility for producing H type unit fragments originating from non-lignin components like aromatic amino acids during pyrolysis [30].

The effect of SE on lignin structure is clearly visible as the relative amounts of pyrolysis products between untreated and pretreated birch samples showed considerable differences (Table 3). In particular, the proportion of pyrolysis products with oxygen in the side chain (vanillin, acetovanillone, 3,5-dimethoxyacetophenone, homosyringic acid, syringaldehyde, sinapyl alcohol, and sinapic aldehyde) was lower in steam-exploded birch in comparison with untreated birch. This indicates the modification of oxygenated functional groups, mainly the β-O-4′ bond, resulting in lower content of pyrolysis products with oxygenated side chains in pretreated birch. Lower contents of oxygen-rich pyrolysis products were obtained from Kraft lignin as a result of the β-O-4′ bond cleavage during Kraft cooking [63]. The increase in the ratio of phenylmethane and phenylethane units to phenylpropane units (Ph-C_1_,C_2_/Ph-C_3_ ratio; Table 3) after pretreatment further supports the cleavage and/or modification of part of the side-chain linkages of lignin substructures and agrees well with the condensed-/pseudo-lignin formation after pretreatment as confirmed by spectroscopy and SEM analysis (see below). It is not surprising that the lignin-derived pyrolysis products of steam-exploded birch and HL are almost identical. The enzymatic treatment is mild and only degrades the polysaccharide fraction.

There was considerable difference between the pyrolysis products of HL before and after AD (Table 3). Notably, the S/G ratio in HL after AD was substantially lower than the original HL, consistent with the S/G ratio reduction also seen by NMR analysis. This could be a result of an increase in G-type pyrolysis products due to the condensed-G-type lignin cleaves off and leaves the normal G-type lignin in the undigested solid material after AD.

### Thermal properties of birch by STA

**Fig. 6:**
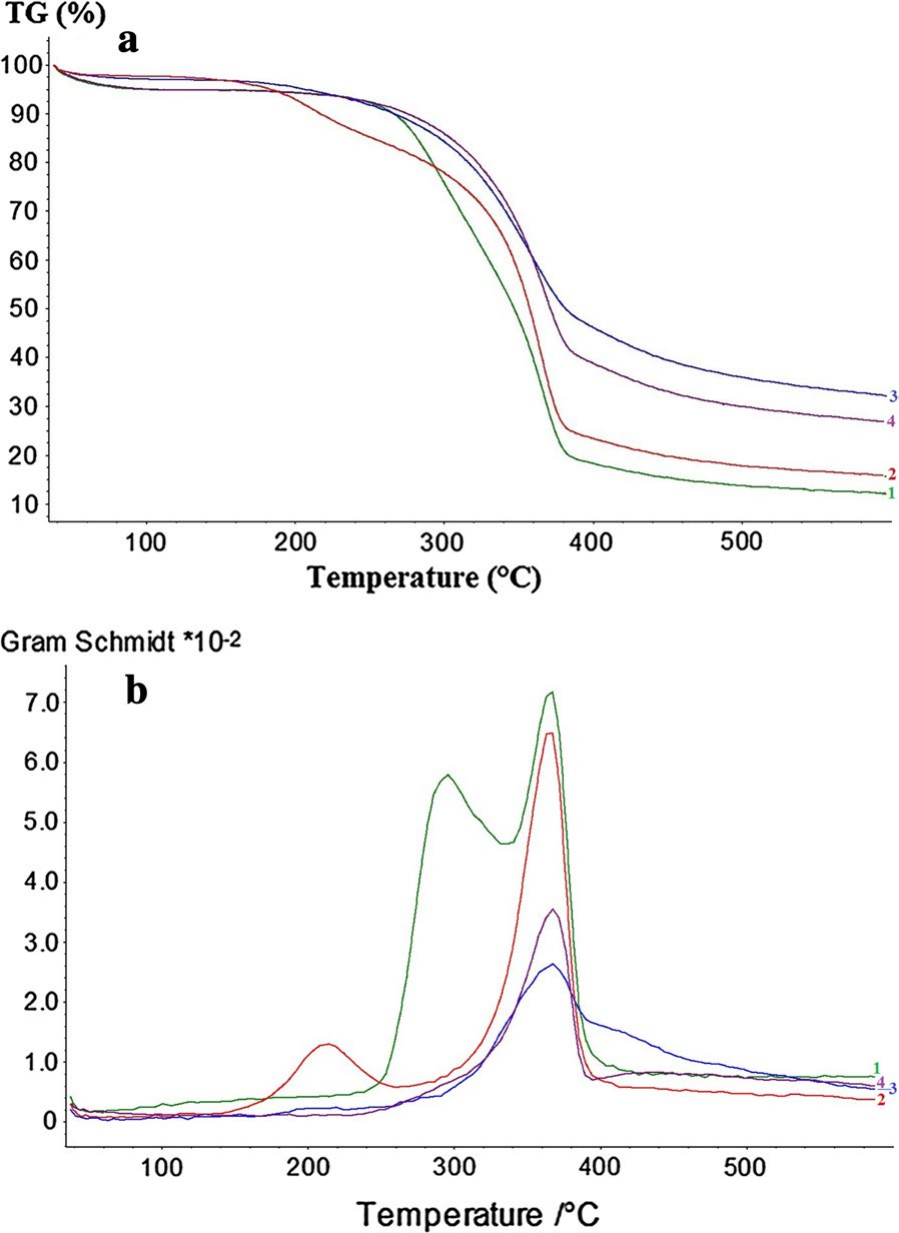
STA analysis of thermal behavior of birch samples. Thermogravimetric (TG) (**a**) and the Gram Schmidt (GS) curves (**b**) of untreated (1), steam-exploded (2), HL (3), and HL birch after AD (4)

Figure 6 shows the thermogravimetric (TG) and Gram Schmidt (GS) curves from the simultaneous thermal analysis (STA) of the various birch samples. The initial slight weight loss for up to 100 °C, mainly seen for the untreated birch and HL after AD, is due to water evaporation (Fig. 6a). The thermal degradation profile of birch in the range of 200–400 °C was different after SE pretreatment, consistent with the changes observed in composition after pretreatment (Table 1). In comparison with untreated birch, the degradation process occurred earlier at a lower temperature for steam-exploded birch; however, more carbonaceous residue remained after 500 °C for steam-exploded birch. This difference is also clearly illustrated in the GS curves, as presented in Fig. 6b. The pretreated birch showed a small mass loss at around 215 °C, which was not observed for the untreated birch. This mass loss could be attributed to the volatile compounds formed from carbohydrates during the steam explosion pretreatment. The maximum thermal decomposition (TGmax) was around 365 °C for both untreated and pretreated birch, but an additional peak around 295 °C was observed in the former (Fig. 6b). It is known that hemicelluloses, cellulose, and lignin thermal degradation occurs in the temperature ranges of 200–300, 275–400, and 200–500 °C, respectively. Therefore, TGmax is attributed to thermal degradation of mainly cellulose and part of lignin. The absence of mass loss at around 295 °C, characteristics to hemicelluloses degradation, after birch pretreatment is consistent with the loss of some of hemicelluloses components during SE pretreatment (Table 1). The observed higher carbonaceous residue after SE pretreatment (TG curve, Fig. 6a) is consistent with the relatively higher content of lignin in pretreated material (Table 1). In general, thermal degradation of pure lignin yields about 30% char, whereas cellulose and hemicellulose yield only about 5 and 5–10% residue, respectively [64].

The effect of enzymatic hydrolysis and AD on the thermal property of HL is clearly shown in the TG and GS curves (Fig. 6). The TGmax for both pretreated birch and HL was observed around 365 °C, but it is lower in the latter due to the removal of cellulose by enzymatic hydrolysis. In comparison with the pretreated birch, the broader TGmax in HL stretching up to 500 °C (Fig. 6b), which shows that the lignin after enzymatic hydrolysis is resistant to thermal degradation. As expected, HL produced higher amount of carbonaceous residue. The TG profile (Fig. 6a) of the HL before and after AD was similar, but higher overall mass loss was observed after AD. The lower amount of carbonaceous residue after AD could be due to the change in lignin structure, particularly the anaerobic degradation of condensed-lignin and pseudo-lignin as confirmed by NMR, FTIR, and Py–GC/MS analyses.

### Morphology characteristics of birch samples by SEM

**Fig. 7:**
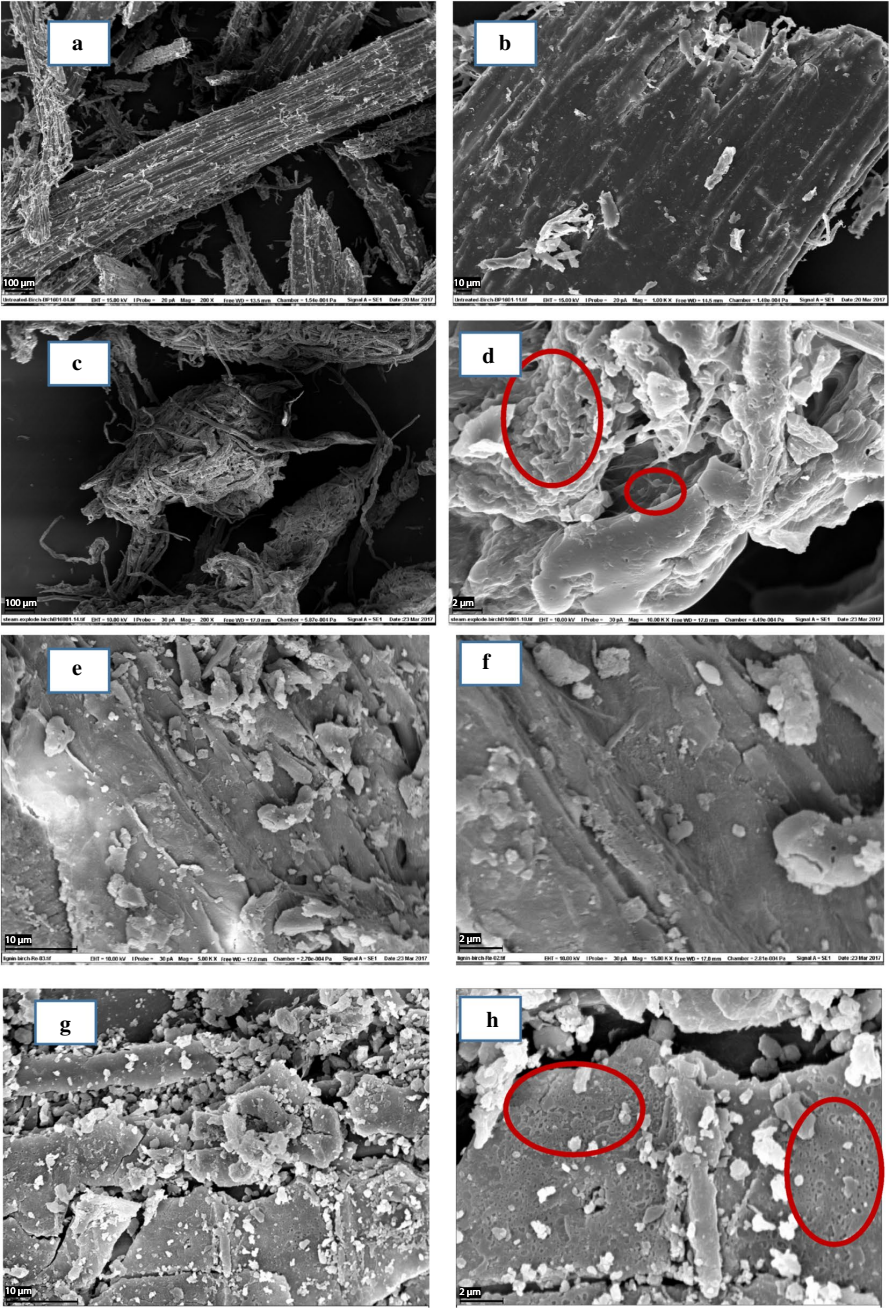
SEM images showing the morphology of birch samples at low and high resolutions. Untreated (**a**, **b**), steam-exploded (**c**, **d**), HL (**e**, **f**) and HL birch after AD (**g**, **h**). The droplets representing lignin re-orientation/pseudo-lignin formation (**d**) and honey-comb-like holes (**h**; may represent microbial attack on the lignin surface) are marked with red circles

The changes in morphology of the birch samples after SE pretreatment, enzymatic saccharification, and AD are presented in Fig. 7. These representative SEM images were taken from a collection of over ten images each for all birch samples at various magnifications. Untreated birch stands out with an intact and smooth surface (Fig. 7a, b). After SE pretreatment, the fibers were broken and formed a porous surface (Fig. 7c, d). Moreover, the higher resolution image of steam-exploded birch revealed formation of droplet on the surface of the material (Fig. 7d), which may be due to the deposition of pseudo-lignin on the surface of the pretreated material [40]. As expected, the major microfibrous cellulose structure was removed and the surface became smooth in HL due to the enzymatic hydrolysis of the carbohydrate component (Fig. 7e, f). Following AD, the HL was broken and formed several honey-comb-like holes on the surface of the material (Fig. 7g, h), which may be attributed to the modification of the surface by anaerobic microbes.

In summary, the NMR and FTIR analysis demonstrated degradation of pseudo-lignin, condensed-lignin, LCC and carbohydrates during AD. Py–GC/MS confirmed the degradation of condensed lignin. The higher weight loss measured by STA for the HL sample taken after AD indicated easier thermal breakdown of lignin deprived of condensed structures. SEM analysis showed that HL was broken into smaller pieces and formed several honey-comb-like holes on its surface, probably a result of microbial attack on HL during its anaerobic degradation. Overall, the degradable lignin structures, LCC, and carbohydrates fractions of HL had contributed to the methane production from AD of HL.

In addition, the chemical analysis and biogas test of steam-exploded birch showed that hemicelluloses removal, lignin relocalization with some structural modification together with broken fiber and porous surface on steam-exploded materials may be responsible for reduction of biomass recalcitrance after SE, which ultimately enhanced the methane production from pretreated birch.

## Conclusions

The lignin-rich material (HL) obtained after enzymatic extraction of carbohydrates has a potential to be used for biogas production. HL birch contained up to 80% lignin with condensed structure due to competing lignin depolymerization (mainly through cleavage of the abundant β-O-4′ inter-unit linkages) and re-condensation reactions during pretreatment. Pseudo-lignin also contributed to the Klason lignin content in HL. The results from biogas potential tests, NMR, Py–GC/MS and FTIR analysis indicated that the carbohydrate fraction and part of the lignin fraction in HL birch were degraded during AD. Pseudo-lignin, condensed-lignin, benzyl ether, and phenylcoumaran were susceptible to AD, whereas β-O-4′ and resinol were resistance to anaerobic microbial degradation. On the other hand, the remaining undigested lignin-rich residues after AD that has less pseudo and condensed-lignin structures and low carbohydrate impurity bears potential for further valorization. This study shows the benefits of using a combination of nylon bag techniques and advanced analytical techniques to study the degradation mechanisms of lignin during AD. It also demonstrated that utilization of HL residues after bioethanol production for biogas production may increase the output of energy carriers from lignocellulosic biomass.

## Additional file

**Additional file 1: Table S1.** Assignments of ^13^C-^1^H correlation signals in the HSQC spectra of the lignin components in birch. **Figure S1.** FTIR spectra of untreated (A), steam-exploded (B), HL (C) and HL birch after AD (D).

## Abbreviations

100Ar: 100 aromatic units (H+G+S)
2D-HSQC NMR: two-dimensional ^1^H-^13^C heteronuclear single quantum coherence nuclear magnetic resonance spectroscopy
AD: anaerobic digestion
BE: benzyl ether linkages
CSTR: continuously stirred tank reactor
DM: dry matter
FTIR: Fourier transform infrared spectroscopy
G: guaiacyl lignin units
GC: gas chromatography
GS: Gram Schmidt
H: *p*-hydroxyphenyl lignin units
HL: hydrolysis lignin
HPAEC-PAD: anion-exchange chromatography with pulsed amperometric detection
LCC: lignin–carbohydrate complex linkages
LMW: lower molecular weight
MWL: milled wood lignin
Ph-C_1_: phenylmethane unit
Ph-C_2_: phenylethane unit
Ph-C_3_: phenylpropane unit
Py–GC/MS: pyrolysis–gas chromatography–mass spectrometry
S: syringyl lignin units
SE: steam explosion
SEM: scanning electron microscopy
STA: simultaneous thermal analysis
TCD: thermal conductivity detector
TG: thermogravimetric
TGmax: the maximum thermal decomposition
VS: volatile solids
