# Supplementary material for: Microbial biogas production from hydrolysis lignin: insight into lignin structural changes
Source: Biotechnol Biofuels. 2018 Mar 9;11:61. doi: 10.1186/s13068-018-1054-7 (PMC5844095; doi:10.1186/s13068-018-1054-7)
Supplement: Supplementary file 1 — Additional file 1: Table S1. Assignments of 13C-1H correlation signals in the HSQC spectra of the lignin components in birch. Figure S1. FTIR spectra of untreated (A), steam-exploded (B), HL (C) and HL birch after AD (D). [file 13068_2018_1054_MOESM1_ESM.docx]

**Additional file**

**Microbial biogas production from hydrolysis-lignin - Insight into lignin structural changes**

Daniel Girma Mulat^a^, Janka Dibdiakova^b^ and Svein Jarle Horn^a^*

^a^Faculty of Chemistry, Biotechnology and Food Science, Norwegian University of Life Sciences, P.O.Box 5003, 1432 Ås, Norway.

^b^Norwegian Institute of Bioeconomy Research, P.O.Box 115, 1432 Ås, Norway

*Correspondence to: Svein Jarle Horn; E-mail: [svein.horn@nmbu.no](mailto:svein.horn@nmbu.no)

**3 pages, 1 Table and 1 Figure are included in the supporting material.**

**Table S1**

Assignments of ^13^C-^1^H correlation signals in the HSQC spectra of the lignin components in birch.

| Label^a^ | *δ*_C_/*δ*_H_ (ppm) | Assignments |
| --- | --- | --- |
| **B_β_** | 53.6/3.04 | C*_β_*−H*_β_* in β-β resinol substructures (B) |
| **–OCH_3_** | 55.6/3.71 | C−H in methoxyls |
| **A_γ_** | 59.5/3.69 | C*_γ_*−H*_γ_* in β-O-4' substructures (A) |
| **C_γ_** | 62.3/3.70 | C*_γ_*−H*_γ_* in β-5 phenylcoumaran substructures (C) |
| **B_γ_** | 71.0/3.88-4.17 | C*_γ_*−H*_γ_* in β-β resinol substructures (B) |
| **A_α_** | 71.9/4.85 | C*_α_*−H*_α_* in β-O-4' units (A) |
| **BE**_α_ | 81.3/4.65 | C*_α_*−H*_α_* in benzyl ether (BE) LCC structures |
| **A′_α_** | 83.1/5.21 | C*_α_*−H*_α_* in *γ*-acylated β-O-4' substructures (A′) |
| **A_β(G)_** | 83.9/4.33 | C*_β_*−H*_β_* in β-O-4' linked to G/H unit (A) |
| **B_α_** | 84.9/4.64 | C*_α_*−H*_α_* in β-β resinol substructures (B) |
| **A_β(S)_** | 85.9/4.11 | C*_β_*−H*_β_* in β-O-4' linked to a S unit (A) |
| **C_α_** | 87.0/5.45 | C*_α_*−H*_α_* in β-5 phenylcoumaran substructures(C) |
| **S_2,6_** | 104.0/6.70 | C_2,6_−H_2,6_ in syringyl units (S) |
| **S′_2,6_** | 106.3/7.31 | C_2,6_−H_2,6_ in oxidized (C*_α_*=O) S units (S′) |
| **G_2_** | 111.1/6.98 | C_2_−H_2_ in guaiacyl units (G) |
| **G_5_** | 114.7/6.71 | C_5_−H_5_ in guaiacyl units (G) |
| **G_6_** | 118.9/6.80 | C_6_−H_6_ in guaiacyl units (G) |

^a^The labels refer to the lignin units and substructures shown in Figures 2 and 3 (main text).


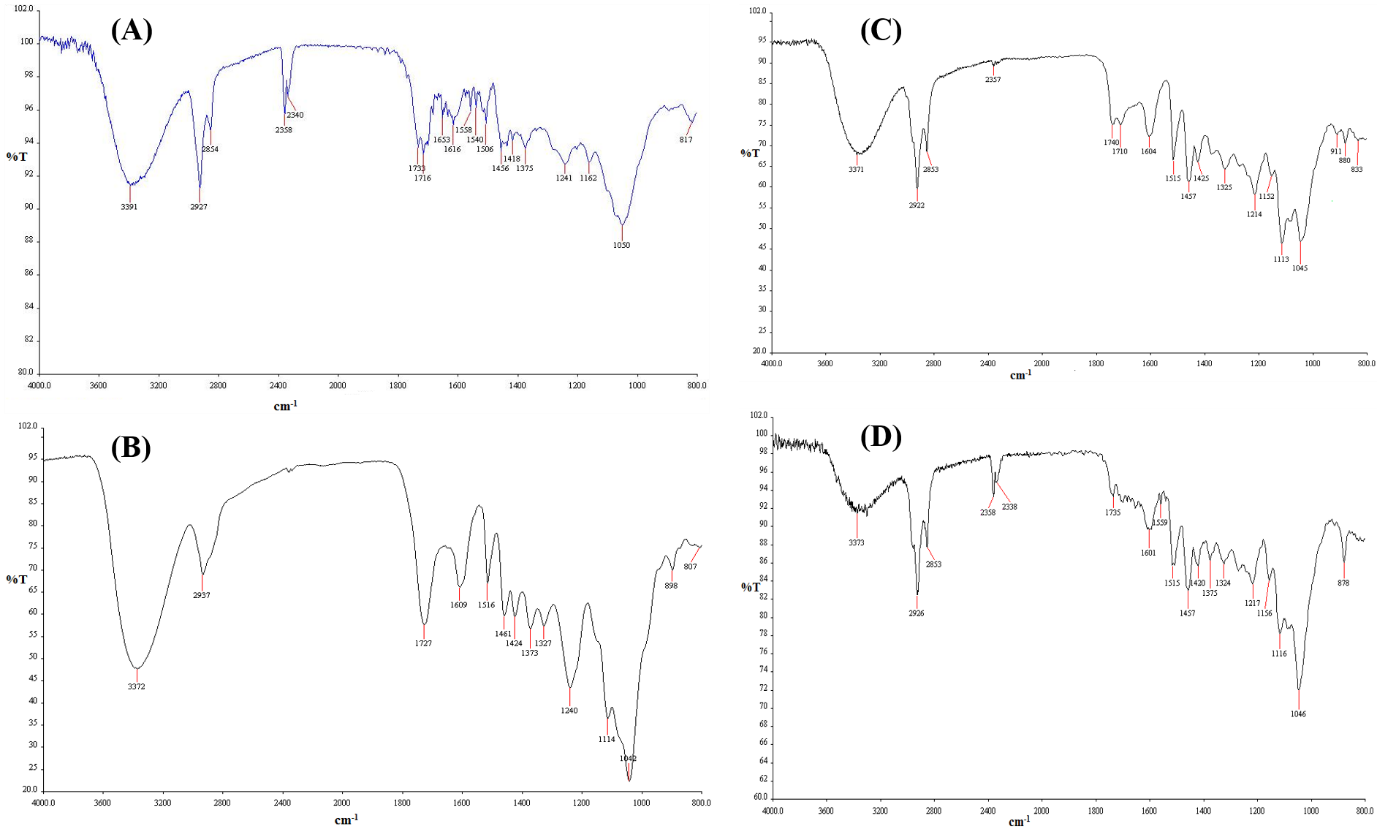


**Figure S1** FTIR spectra of untreated (A), steam-exploded (B), HL (C) and HL birch after AD (D).
